# OpenFlume: An accessible and reproducible benchtop flume for research and education

**DOI:** 10.1016/j.ohx.2024.e00583

**Published:** 2024-09-19

**Authors:** Maya Lewis, Eli Silver, Robert Hunt, Daniel M. Harris

**Affiliations:** Brown University, School of Engineering, 184 Hope St, Providence, RI 02912, United States of America

**Keywords:** Flume, Flow tank, Drag, PIV, Open-source

## Abstract

Open-channel flumes are an important tool in fluid mechanics research and education. However, the few commercially available small-scale flumes are generally expensive and lack complete characterization. In this work, we present an open-source, low-cost, modular benchtop laboratory flume that is designed to be accessible and reproducible. The flume is assembled from widely available materials and hardware, and fabricated exclusively using tools and machinery commonly found in workshop spaces. The recirculating water flow through the system is driven by a controllable electric coolant pump designed for automobiles. Our design has a flow cross-section of 5 × 5 cm, adjustable flow velocity, and a modest overall footprint. All design files and build instructions are shared in a digital repository ensuring openness and reproducibility. The flow in the test section is characterized using particle image velocimetry (PIV) and is shown to be of high uniformity with low turbulence intensity. Furthermore, direct measurements of the drag force on a submerged sphere are reported for a range of control parameters, and exhibit good agreement with established empirical values.

## Graphical abstract

**Figure:**
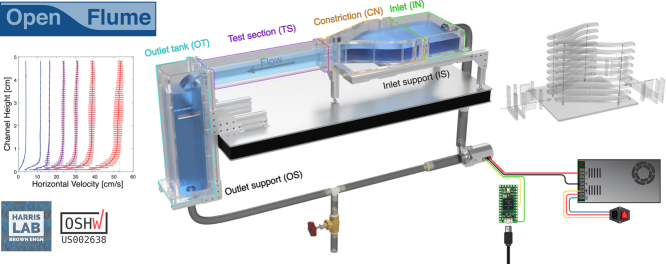

**Table:** 

Specifications table	

**Hardware name**	OpenFlume
**Subject area**	•Engineering and material science
**Hardware type**	•Mechanical engineering and materials science
**Closest commercial analog**	EmFlume1 Hydraulic Flume (Emriver, Inc.)
**Open source license**	Creative Commons Attribution-ShareAlike 4.0 International (CC BY-SA 4.0)
**Cost of hardware**	$ 1079.66
**Source file repository**	https://doi.org/10.5281/zenodo.13312832
**OSHWA certification UID**	US002638

## 1 Hardware in context

Flow tanks, or flumes, are used for experiments in various fields — from geological and environmental applications studying sediment transport, to biomechanical studies of biologically inspired swimmers, to civil and naval applications studying the flow properties caused by submerged structures, among countless others [1], [2], [3], [4]. As a consequence of this remarkable breadth of applications, flumes also commonly appear in undergraduate education [5]. While many research questions demand large-scale flume facilities (e.g. meter scale or larger), smaller-scale flumes are more cost-efficient with less demand on infrastructure, and are also better suited for addressing certain research questions naturally situated at smaller length scales, such as swimming at intermediate Reynolds number [6], [7], [8] or capillary–gravity wave drag [9]. OpenFlume was born from Harris Lab’s desire to study drag forces created by flow past a partially submerged sphere at the capillary scale (millimeter to centimeter scale), where surface tension effects remain important. Due to the relatively small scale of the research question to be addressed, a bench-top scale flow tank was needed to perform the desired experiments [10]. At the time, the few existing commercially available flumes at this scale were both costly (on the order of $10k) and lacked detailed characterization, motivating the development of a bespoke alternative.

From these considerations and constraints emerged OpenFlume — tackling the question of how we can design and construct a flume both suitable for our experiments and reproducible for others with similar experimental needs. For educational purposes, we see the low cost and customizability of OpenFlume as primary benefits, whereas the careful flow conditioning and characterization make it additionally suitable for certain research applications as well. While the current version of the flume was designed and characterized for applications related to drag on centimeter-scale bodies, the openness and deliberate modularity of the OpenFlume will readily facilitate extensions into other related fields such as environmental flows.

## 2 Hardware description

OpenFlume is a low-cost open-source benchtop flow tank capable of providing controlled and conditioned uniform flows for fluid dynamics experiments, demonstrations, and education. The flume, fabricated exclusively using tools and machinery commonly found in workshop spaces, was explicitly designed to be accessible and easily reproduced.

**Fig. 1:**
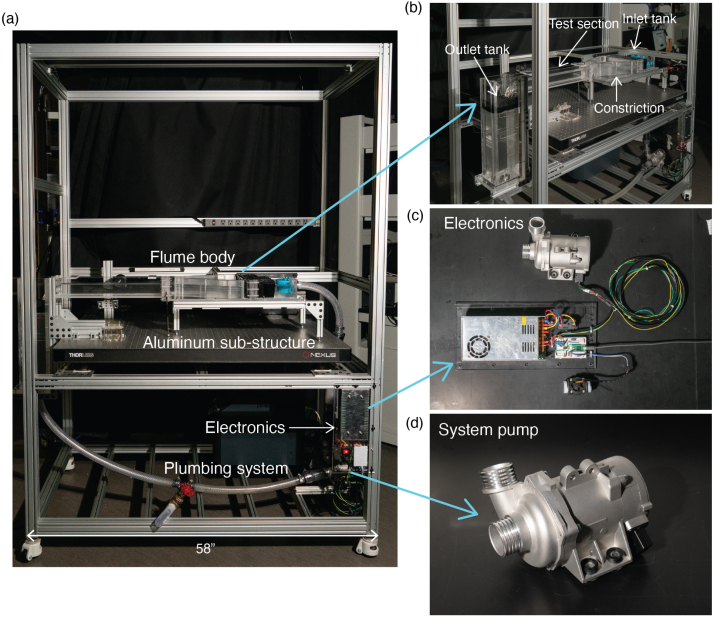
(a) Overall OpenFlume system with the four major components labeled: flume body, aluminum sub-structure, electronics, and plumbing system. (b) Flume body overview, with the four major subassemblies labeled: inlet tank, constriction, test section, and outlet tank. (c) Electronics system. (d) Water pump used in flume system.

There are four main components of the flume: the flume body, the supporting aluminum sub-structure, the plumbing, and the electronics. The overall OpenFlume system and key subsystems are shown in Fig. 1.

### 2.1 Flume schematic

The body of the flume is composed of four main sections: the inlet tank, the constriction, the test section, and the outlet tank as shown in Fig. 1(b). The water violently enters the inlet tank where it passes through a sequence of flow conditioning measures designed to break-up large eddies and unidirectionalize the flow — an initial inlet diffuser, mesh gates, and a flow straightening honeycomb structure. Once through the inlet section, the water enters the constriction where the width of the channel is reduced in order to minimize the boundary layer thickness on the walls in the test section [11]. The constriction curve is a fifth-order polynomial curve designed using Rhinoceros 3D Software enforcing continuity of curvature at the endpoints. The water then enters the test section at which point the flow is straightened and the boundary layer thickness is small. The water exits via the outlet tank where it passes through filters and a diffuser plate meant to prevent extraneous particles from entering the pump as well as minimize the recirculation of entrained air. The flume is a modular system in the sense that each of these four main sections are separate entities connected by a standard flange that can individually be removed, repaired, or redesigned to meet specific needs without having to rebuild the entire flume body.

The flume body is supported by an aluminum sub-structure frame composed of 80/20 aluminum extrusions and hardware. This sub-structure is adaptable to the needs and constraints of our given setup. Our flume and support structure sit on an optical breadboard that allows for parts of the support structure to be directly bolted into the table for additional support. These structures can be seen in Fig. 1(a).

The water is recirculated via a common electric water pump (TEMSONE 11517586925) meant for automobile engines (Fig. 1(d)). The pump is controlled by a microcontroller (Teensy 4.0) that interfaces with the pump using a circuit built on a solderless breadboard (Fig. 1(c)). The microcontroller also reads and reports the volumetric flow rate measured by an inline flow meter (DIGITEN FL-1208). The plumbing of the system is made up of vinyl tubing and various standard fittings. The plumbing system is also equipped with an exit valve that can be used to quickly drain the flume.

## 3 Design files summary

All design files needed to build and or customize OpenFlume are available via digital repository. The design files include all CAD files for the build, a complete parts list made using Rhinoceros 3D, a full circuit diagram, all necessary software, and assembly videos. All CAD files have prefixes that designate the section they are used for: IN- indicates files used for the inlet tank, CN- refers to the constriction, TS- refers to the test section, OT- refers to the outlet tank, IS- refers to the portion of the aluminum sub-structure that supports the inlet tank, constriction, and test section, and OS- refers to the portion of the aluminum sub-structure that supports the outlet tank. The assembly videos and assembly drawings were all generated using the same CAD files provided in the repository. The parts list is meant to give an overview of all the pieces to be cut as well as suggestions for how to break down stock materials to minimize waste. Refer to Table 1 for a summary of all design files.

## 4 Bill of materials summary

The Bill of Materials is also available via the digital repository. The materials are organized by what system they are used in: the flume body and its construction, electronics, plumbing, and aluminum support sub-structure. We have also included a tool list that outlines all of the tools we used in the construction of OpenFlume.

## 5 Build instructions

**Table 1:** Summary of design files available in the repository.

Design filename	File type	Open source license	Location of the file
Flume body design	CAD files	CC BY-SA 4.0	digital_repository
Assembly videos by section	Videos	CC BY-SA 4.0	digital_repository
Dimensioned assembly drawings	.pdf	CC BY-SA 4.0	digital_repository
Complete parts list	.3df .dxf	CC BY-SA 4.0	digital_repository
Electronics design files	.kicad_pro	CC BY-SA 4.0	digital_repository

### 5.1 Design methodology

The intent and motivation driving OpenFlume was to build a flume that was experiment-ready, reproducible, and customizable. The modular design of the flume body was implemented for both ease of construction, use, and maintenance. The modular design of the system also readily enables future customization based on the needs of a specific project. For example, the test section could be longer or deeper based on flow needs in the window of investigative interest, without fully changing the entire design or the build instructions.

In order to ensure reproducibility and increase accessibility, we limited ourselves to a set of accessible and readily available tools. All of the parts of the flume body can be cut and machined using machines common in workshop spaces: a table saw, router table, and drill press. While we opted for manually operated machines for most of our cuts, namely for the flume body parts, these cuts could also be made using a CNC router, which would save time when fabricating the flume body. However, the main reason for using manual tools rather than a CNC router was to limit our toolset in the interest of accessibility, as our chosen toolset is less expensive and more common than CNC routers. This decision forced us to simplify our design as much as possible to avoid unnecessary intricacies that would not be possible to reproduce using manual tools or without other more advanced techniques.

A principal motivation behind avoiding using a laser cutter for the flume body parts was to avoid surface cracking, otherwise known as crazing, an effect common when laser cutting acrylic where the internal thermal stress caused by the laser cutter leads to small cracks along the cut edge that grow and bifurcate over time [12]. This effect is worsened when the edges are subject to certain chemical compounds, such as the acrylic solvent used to weld edges, which exacerbates the crazing. In previous iterations of our flume [10], the flume body was constructed of 5.5 mm thick acrylic rather than 9 mm acrylic and the pieces were all laser cut. At the thinner acrylic thickness, the crazing along the edges resulted in leaks at joints. For the final iteration presented here, we switched to a thicker acrylic, avoided laser-cutting pieces that would need to be bonded using solvent glue, and added internal braces to reinforce joints. All of these adjustments contributed to a leak-proof final design. The thicker acrylic also worked to make the flume more rigid, as the weight of the water across the flume makes the flume susceptible to bending and warping under the water’s weight. We laser cut some non-structural pieces of the flume, such as the accessories for flow control and conditioning and certain jigs helpful for assembly. However these parts could alternatively be cut using a combination of the same manual tools used for the flume body or a CNC router. The flume can thus be built with a range of access to tools and manufacturing skill levels.

The recirculating flow system was designed around an electric water pump for cars. When considering a pump for the system, we needed one that had variable speed, was electric, and was able to provide us with the desired range of flow rates. The selected pump is readily available on Amazon and other online retailers and is inexpensive compared to other water pumps meant for experiments.

### 5.2 Fabrication tools

**Caution: Throughout the build process, protect yourself with appropriate PPE and review safety procedures and instructions before using any tools or listed components.**

#### 5.2.1 Overview of tools and machines

To fabricate the body of the flume, we used various machines common in woodshops fitted with acrylic-specific cutters. Most cuts to rough size were made using a table saw fitted with a plastics cutting blade. We also used a drill press with drill bits designed for acrylic that minimized chips and cracking within the acrylic. Various fabrication jigs and templates, and flow conditioning and control devices were cut on a laser cutter, but they could also be made using a combination of the table saw, router table, and drill press.

#### 5.2.2 Making fabrication jigs and templates

We designed various jigs and templates to assist with accurate alignment when welding, drilling, and routing pieces of the flume body. Jigs refer to tools used to hold parts in place, typically during welding or gluing, while templates refer to stencils to guide in routing or drilling. Table 2 summarizes these various fabrication tools, detailing their material and use. All necessary CAD files to fabricate jigs and templates can be found in the repository. Please note that while we fabricated these parts using a laser cutter, they could also be machined using a CNC router or a set of manual tools. Note that all of the CAD files are sized to the exact dimension of the system: for laser-cut pieces it is important to compensate for the laser cutter’s ablation width or kerf when exporting the files to be laser cut to ensure the cut piece matches the piece in the model as accurately as possible.

**Table 2:** Fabrication jigs and templates guide.

Fabrication jig name	Material	Use	Quantity
Test section flange drill template	5.5 mm acrylic, 3 mm acrylic, acrylic adhesive	Aligning flange holes for bolts for test section flanges (TS-1, TS-2)	1

O-ring groove jig	1/4" MDF or 5.5 mm acrylic	Routing o-ring groove into test section flange (TS-F1)	1

Test section alignment jig	5.5 mm acrylic	Aligning test section walls (TS-S1, TS-S2) when welding to ensure consistently spaced/wide channel	2

Bulkhead fitting hole template	1/4" MDF or 5.5 mm acrylic	Routing hole in parts (OT-B, IN-S3) for bulkhead fitting at the inlet and outlet	1

Outlet flange template	1/4" MDF or 5.5 mm acrylic	Routing flange shape outlet tank wall (OT-S3)	1

Outlet alignment jig	5.5 mm acrylic	Maintain even spacing/width when welding up the outlet tank	2

Inlet alignment jig	5.5 mm acrylic	Maintain even spacing/width when welding up the inlet tank	2

Constriction alignment jig	5.5 mm acrylic	Maintain constriction shape when welding up constriction	1

Constriction curve routing template	5.5 mm or 9 mm acrylic	Routing rough cut constriction curves to final dimensions	1

Test section flange routing template	1/4" MDF or 5.5 mm acrylic	Routing test section flanges to shape (CN-F2, TS-F1, TS-F2)	1

Inlet flange routing template	1/4" MDF or 5.5 mm acrylic	Routing IN-F1 and CN-F1 flanges to shape from initial rectangular cut	1

Inlet flange drill template	5.5 mm acrylic	Transferring center punches for inlet flange holes for drilling	1

### 5.3 Acrylic bonding procedures

For welding the acrylic pieces, we used an acrylic solvent, Weld-On 4, a product containing Methylene Chloride and Trichloroethylene [13]. There are various techniques necessary for effectively bonding the acrylic pieces to achieve precise joints. It is important to note that the working time of Weld-On 4 is very short, between 1–2 min. This specification means that you have less than two minutes to align and adjust your glue-ups and fully clamp before the solvent has cured — necessitating plenty of preparation and planning, and often an extra set of hands. It is also important to note that Weld-On 4 is considered a hazardous material, and proper safety precautions should be made when using the product such as working in a fume hood or well-ventilated area and wearing safety goggles and nitrile gloves. There are two main cases for welding that we implemented: butt-joint welding and face welding. The butt-joint welding we implemented for most of the flume body involved welding edges of the acrylic together to form joints. In order to bond the curved constriction pieces into the completed lamination, we implemented a slightly different approach for welding faces with significant contact surface areas. Below are the procedural steps for completing glue-ups. For all glue-ups, we used a variety of clamping methods determined by the needs of each joint and the equipment available to us, mainly bar clamps.

*For all welding cases (butt-joint and face):*

1. Before all glue-ups, ensure that your clamps are handy and fully opened. Because the working time of Weld-On 4 is very short, you want to avoid wasting any time once the solvent has been applied.
2. Next, do a dry run of the glue-up to ensure you have all of the necessary parts and that they are handy. Place the parts where you plan to weld them as a test run and ensure the configuration matches the design. Create a plan for clamping that ensures the part will be stable once clamps are applied.
3. Once the pieces are placed where you plan to weld them, cut the protective film of the acrylic where the edges will meet using a craft or utility knife, leaving the protective film everywhere else. Only the surfaces that will be bonded should be exposed — this step helps to keep the welding precise while minimizing defects on surfaces caused by solvent drips.
4. Clean the surfaces to be bonded with a low-lint delicate wipe and clean compressed air if necessary. If there is any significant dirt or oil present on the surfaces, use soap and warm water to clean. Avoid using isopropyl alcohol (IPA) or other solvents to clean the surface as IPA can cause acrylic edges to craze, causing cracks to form and spread along the edges of acrylic. It can be helpful to scuff the polished surfaces with fine sand paper to increase surface area for solvent to act on, however surfaces should then be cleaned to avoid embedding of abrasive particles generated during sanding.

*Steps for butt-joint welding:*

**Fig. 2:**
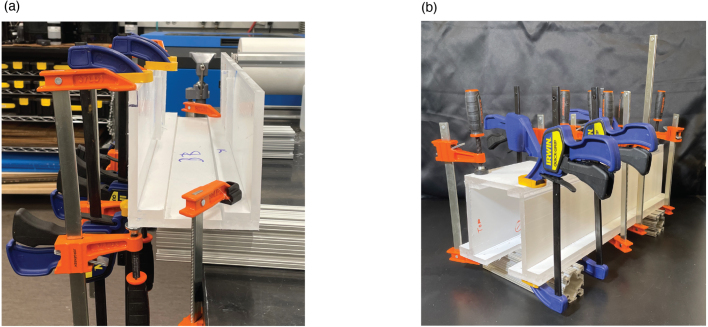
(a) Example of clamp placement for welding the outlet tank. The clamping and welding are forming the joint between OT-S1 and OT-B. (b) Example of clamp placement for the final stage of welding the outlet tank when OT-S4 is welded to OT-S1 and OT-S2, completing the box-like shape. Note that a large number of clamps are used and they are distributed uniformly and symmetrically throughout the piece, applying adequate pressure to the joints being welded.

1. Gather the pieces to be welded and join the pieces at a right angle to form the desired joint, using a gluing jig if necessary. Using a needle-tipped applicator bottle (these often come with Weld-On products), run the applicator along the seam of the desired joint, letting the glue wick into the joint.
2. Weld-On 4 has a working time of 1–2 min during which you can make slight adjustments to ensure alignment, using welding jigs if necessary.
3. Loosely apply the clamps to the joint being bonded. The clamps should be initially tight enough that the clamps hold the structure together but loose enough that with slight force, the pieces can be slightly nudged. Within 1–2 min of the solvent being applied, make any necessary slight adjustments to the pieces being bonded. After that window, fully tighten clamps, ensuring pieces do not shift or deform during final tightening. Example clamp arrangements are shown in Fig. 2.
4. Once clamps are placed and tightened, and before the solvent cures, clean solvent squeeze-out from joints by cutting a plastic straw at an angle and running the angled straw tip along the welded joint.
5. Leave the clamps on the joint for 20-30 min before removing.
6. The times outlined above are specific to the product we used, other products will inevitably have different specifications. Follow solvent manufacturer instructions for working time, clamping time, and time to fully cure the joint.

*Steps for face welding:*

For glue-ups that involve large surface areas and entire faces being bonded (rather than just edges), the technique varies slightly to ensure sufficient and even welding with minimal trapped bubbles. This is most relevant for welding the constriction curves together but can be applied where needed when the surface area of the welding surface seems wide enough that the standard wicking application will not span the width of the welding surface.

1. For constriction curves, begin by gathering the pieces to be welded. Identify a base piece that will be fixed during the welding and a welding piece, the piece that will be welded onto the base piece. Install the 1/4” alignment dowels into the base piece and then install the welding piece on the alignment dowels, slightly separating the faces of the base piece and the welding piece to create a consistent gap between the surfaces.
2. On the base face, apply the solvent directly on the face all over. For glue-ups on large surface areas, this method is more effective than wicking along the seams, as for wide areas the wicking cannot reach the full span of the face.
3. Let the acrylic solvent sit on the face for 30–45 s, allowing the solvent to dissolve the acrylic surface.
4. Place the curve to be bonded on the welded face and loosely apply clamps.
5. Align the gluing faces. Once faces are aligned, fully tighten clamps ensuring even pressure. Additional clamping cauls should be used to distribute pressure over large surfaces to keep the welded pieces from deforming under local clamping loads.
6. Once clamps are placed and tightened and before the solvent fully cures, clean squeeze-out from joints using the blade of a utility or craft knife.
7. Leave the clamps on the joint for 20–30 min before removing.

**Fig. 3:**
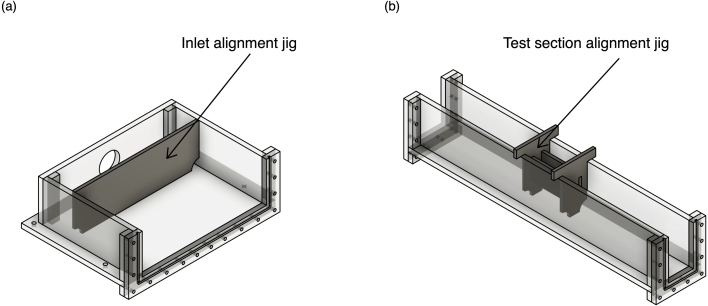
(a) Example of how to position and use the inlet alignment jig to achieve desired and consistent dimensions when bonding the inlet tank. (b) Example of how to position and use the test section alignment jig to achieve desired and consistent dimensions when bonding the test section.

As previously mentioned, there are multiple welding jigs designed to ensure alignment for the glue-ups. See the diagrams in Fig. 3 that demonstrate the orientation of the welding jigs for their given sections. We suggest dry-clamping joints involving jigs to make sure pieces can be properly aligned prior to applying solvent. Once alignment is satisfactory, follow the welding procedures previously outlined.

### 5.4 Routing-to-template procedure

For multiple elements of the flume body, such as the flanges, rough-cut pieces must be cut to their final geometry using a router table. We used a standard procedure for routing these parts that involved using a routing template specific to the piece’s geometry. Section 5.6 details procedures for specific parts, but here we will first outline general procedures that can be applied to all cases of routing pieces to size using a router template.

Begin by securing the cut-to-rough-size part to the laser-cut routing template. To secure it, place a patch of masking tape on the acrylic piece and a patch of masking tape on the template. Apply super glue to the patches of masking tape and glue the two pieces together. By using the masking tape and only applying glue to the masking tape, you can safely rout the acrylic piece while keeping the template secure as well as remove the acrylic part from the template after routing is complete without causing damage to either. Ensure alignment between the part and the routing template when securing the two. Once the template is secured to the acrylic piece, equip the router table with the bottom-bearing flush trim router bit. Place the piece on the router table with the routing template on the bottom and the acrylic piece on top. Position the router bit so that the bearing will roll along the template and the blade cuts the acrylic piece. Turn on the router and slowly move the piece along the template clearing out the material until the bearing of the bit reaches the routing template. Make sure to feed the material into the router blade in the correct direction (conventional not climb cutting) which can be counterintuitive when routing inside features. Follow the template, keeping consistent pressure against the bearing throughout the process.

### 5.5 Acrylic cut sheet

Except for the curved constriction pieces and the weir spacer flange, all acrylic parts of the flume body were cut on a table saw using a blade specifically made for cutting plastic. In the digital repository, there is an overall cut list made using Rhino3D. This file contains all the parts that need to be cut from the necessary stock material. One layer of the file indicates the raw stock material, whether it be 9 mm acrylic or 5.5 mm acrylic. The blue layer outlines the specific parts and corresponds to a designated part name that matches the CAD files. For cuts of the flume body made using the table saw, there is a layer that indicates suggested rip cuts, providing a rip guide to efficiently break down the stock material. When using the rip guide, it is important to maintain a factory-cut reference edge that is a known straight edge. This reference edge should be positioned against the table-saw fence for each cut to ensure any errors when cutting are not compounded. The flume body pieces could alternatively be cut using a CNC machine — the preceding welding procedures and assembly guide in the subsequent section would be unchanged.

The curved constriction pieces were not cut on the table saw but were instead laser-cut to an oversized dimension and routed to the design dimensions. The curved constriction pieces also could be rough cut to oversized dimensions using a combination of table saw and band saw.

Leave all protective film on cut acrylic pieces to minimize scratches and damage.

### 5.6 Flume assembly guide

**Fig. 4:**
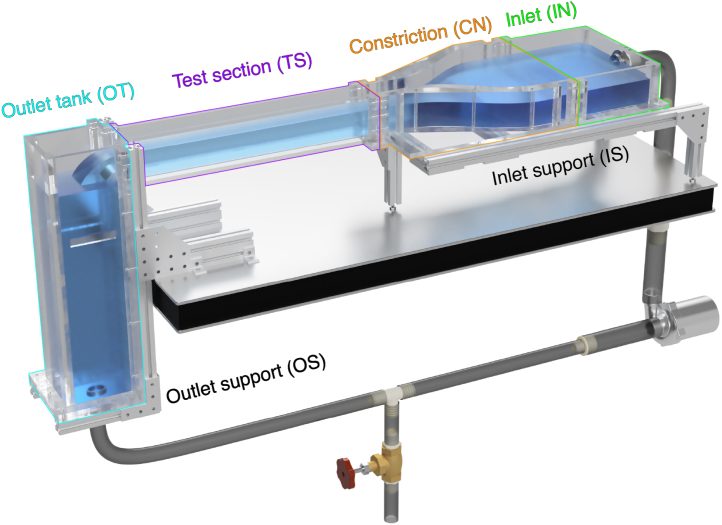
Rendering of the completed flume with each sub-assembly labeled and outlined.

For each section of the flume body, we have put together instructional videos available in the digital repository that demonstrate the intended sequence for welding pieces. In the subsequent sections of this assembly guide, we will show a summary of these assembly instructions via drawings that show the assembled section, the part numbers needed for the section, and a suggested assembly order for the glue-ups. The subsequent section will also detail any unique techniques required for any of the parts in each sub-assembly. After assembling, we sanded pieces as necessary — finely sanding constriction pieces that had excessive squeeze-out and coarsely sanding flange faces that were fouled with solvent during construction. An overall CAD rendering of the flume with all subassemblies is shown in Fig. 4.

#### 5.6.1 Inlet tank (IN)

**Fig. 5:**
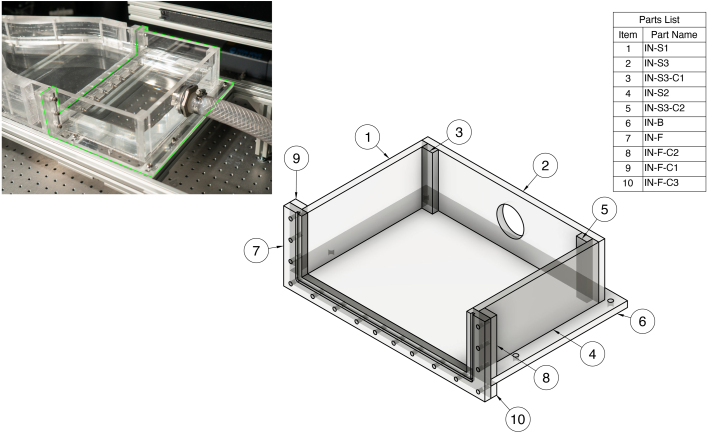
Assembly drawing overview for inlet tank (IN).

The inlet tank is composed of three sides, a base, a flange, and supports for these components. All inlet parts will be labeled with the prefix “IN-” in the CAD files. The inlet tank and corresponding parts list are shown in Fig. 5.

In order to create the hole in part IN-S3 for the bulkhead fitting to enter, you must rout the hole using the bulkhead fitting hole template previously discussed in the fabrication tools section (Section 5.2) and following procedures outlined in the routing-to template-section (Section 5.4). Secure the cut-to-size IN-S3 part to the bulkhead fitting hole template using the temporary gluing method in Section 5.4. Once the template is secured to the acrylic piece, drill a hole through the template hole with a diameter larger than that of the bottom-bearing flush trim router bit. Once an initial hole is made for the router bit to fit through, follow the routing-to-template procedures to achieve the desired geometry of IN-S3.

To create the shape of the inlet flange for part IN-F, use the inlet flange routing template. Follow the procedures outlined in the routing-to-template Section 5.4. Once the inlet flange (IN-F) is shaped, you must rout the O-ring groove for the piece. To rout the inlet O-ring groove, secure stops at the router table at the distances outlined in Fig. 8(a). Equip the router table with a 1/8” straight router bit and place part IN-F on the router table. Adjust the height of the tool to ensure the bit is exposed by 3 mm from the base face. Turn the router on and push the acrylic piece towards the router tool using the fence on the right side as a guide. Once the acrylic piece hits the perpendicular fence, move the acrylic piece to the left now using the upper fence. Once the piece cannot move any farther to the left, push the acrylic piece down using the left side fence as a guide. Now the piece has an O-ring groove. Complete one more pass using this process to ensure the groove is clear.

#### 5.6.2 Constriction (CN)

**Fig. 6:**
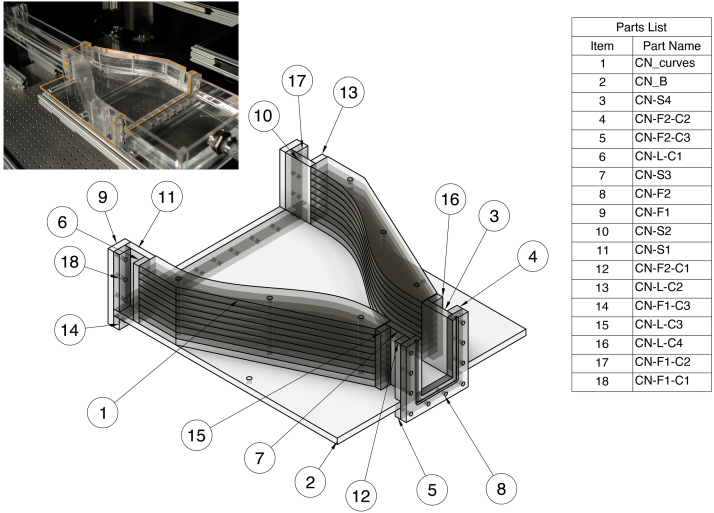
Assembly drawing overview for constriction (CN).

The constriction is composed of a base, curved constriction walls, two flanges (one connecting the constriction to the inlet tank and one connecting the constriction to the test section) and additional walls connecting the curved wall to the flanges. The curved constriction wall is constructed by cutting nine constriction curved pieces to size (per side) and face welding them together one at a time to create a curved wall. All constriction parts will be labeled with the prefix “CN-” in the CAD files. The constriction and corresponding parts list is shown are Fig. 6.

Both constriction flanges must be routed to shape. To rout the constriction flange that connects to the inlet tank, CN-F1, use the inlet flange routing template. To rout the constriction flange that connects to the test section (CN-F2) use the test section flange routing template. For both flanges, follow the same routing-to-template procedure outlined in Section 5.4.

The constriction curves are fabricated by first laser cutting the acrylic to a rough size (slightly larger than the final piece’s dimensions) and then using the constriction curve routing template to rout the pieces to their final size. First, laser cut the constriction curves to an oversized dimension using the Rhino3D constriction template. The lasercut file includes drill point markers at each drill location. Once lasercut, drill the holes in each constriction curve as indicated by the lasercut drill point markers using the 1/4” acrylic drill bit. Once the holes are drilled, the constriction curves must be routed to their final dimensions. To do so, follow the same procedures outlined in the routing-to-template Section 5.4 specifically using the 1/4” alignment dowels to ensure proper alignment and the constriction curve routing template. Repeat for all constriction curves.

For welding the curve constriction pieces together, follow the face-welding guide detailed in Section 5.3.

Once all of the constriction curves are bonded, and before welding the constriction curved wall to the base, you must sand the curved faces of the constriction curve. We began sanding at 60 grit and incrementally worked our way up to 1000 grit. It is important to maintain the shape of the curve when sanding. To maintain the curve, we used a hand sanding block with a curved back. We placed the sandpaper around the curved side of the sanding block and were thus able to sand with a radius to maintain the geometry of the constriction curve when sanding. After sanding the curves of the constriction, use the plastic polish set to finish the curved face. Begin with the 3 solution (coarsest grit), move to the 2 solution next, finally followed by the 1 solution (finest grit). Apply the solution to a microfiber rag and rub the curved surface using a small circular motion.

Once the constriction curves are bonded and sanded, you can begin welding the entire constriction assembly.

#### 5.6.3 Test section (TS)

**Fig. 7:**
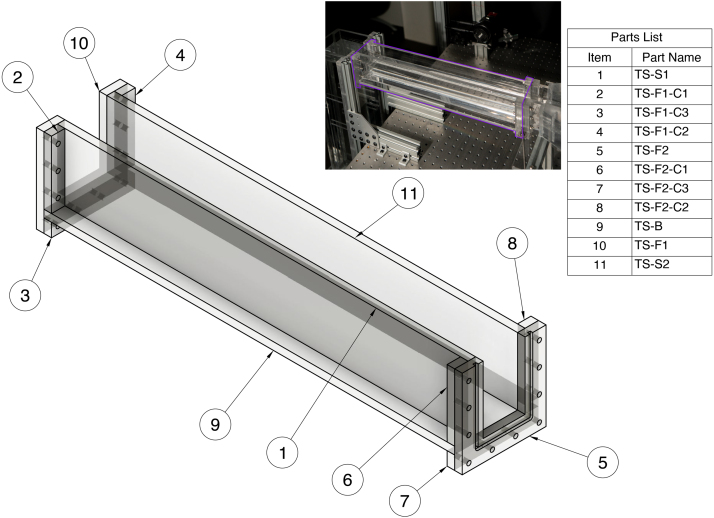
Assembly drawing overview for test section (TS).

The test section is composed of a base, two sides, two flanges (one connecting to the constriction and one connecting to the outlet tank), and brace structures for those features. All test section parts will be labeled with the prefix “TS-” in the CAD files. The test section and corresponding parts list are shown in Fig. 7.

To rout the test section flanges (TS-F1 and TS-F2) to shape, use the test section flange routing template and the procedure outlined in Section 5.4.

**Fig. 8:**
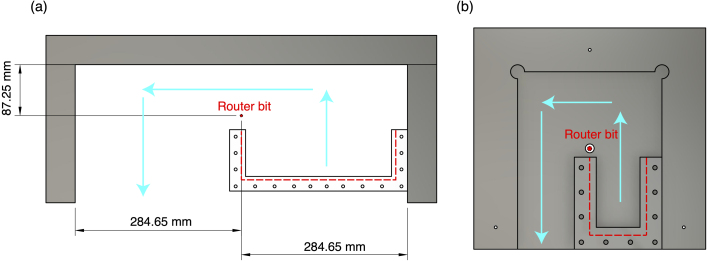
(a) Guide for arranging stops for routing O-ring groove in inlet flange. The outlined distances indicate the distance of stops from the center of the router bit. The red circle represents the router bit location while the blue arrows indicate the path of the piece through the router. (b) Guide for routing test section O-ring groove in the test section flange. This shows the orientation of acrylic flange piece and O-ring groove routing jig when looking down at router table. The red circle represents the router bit location while the blue arrows indicate the path of the piece through the router.

Once the TS-F1 piece is routed to its flange shape, you can rout the O-ring groove. To create the O-ring groove in TS-F1, you must use the O-ring groove jig. The O-ring groove jig is composed of two faces: the back face (the face that is a solid square with one circle cut out of the middle) and the guide face (the side with the pattern). To rout the O-ring groove using the jig, begin by equipping the router table with a 1/8” straight router bit. Place the routing jig on the router table top with the back face facing down and the guide face facing up with the router bit tool sitting in the center hole of the jig. Clamp the routing jig to the router table top ensuring the jig is secure and will not shift when pressure is applied. Begin with the cut flange piece in the configuration shown in Fig. 8(b).

Adjust the height of the tool to ensure the bit is exposed 3 mm from the base face. Turn the router on and push the acrylic piece towards the router tool using the right side of the jig as a guide or fence. Once the acrylic piece hits the perpendicular wall of the jig, move the acrylic piece to the left now using the upper side of the jig as a fence. Once the piece cannot move any farther to the left, push the acrylic piece down using the left side of the jig as a fence. Now the piece has an O-ring groove. Complete one more pass using this process to ensure the groove is clear.

After the flange parts (TS-F1 and TS-F2) have been shaped on the router, and TS-1 has its O-ring groove, begin welding the assembly. Once all parts are bonded, drill the flange holes so the holes also extend to the brace structures. Sand the seams of the section, starting at 60 grit and working up to 220 grit.

#### 5.6.4 Outlet tank (OT)

**Fig. 9:**
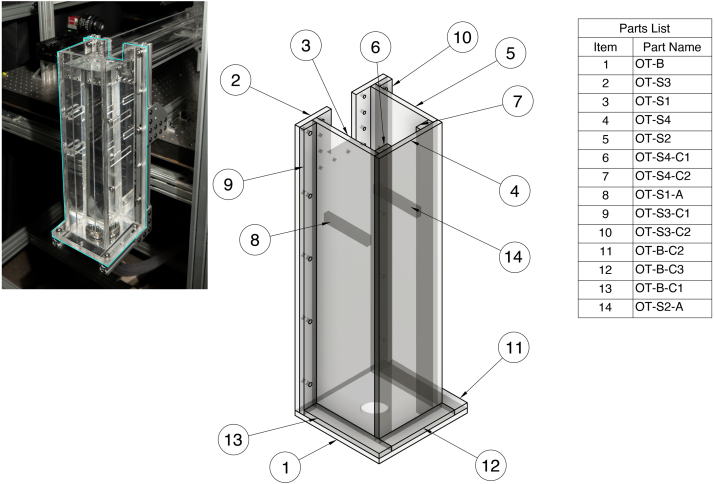
Assembly drawing overview for outlet tank (OT).

The outlet tank is composed of a base, four walls, a weir spacer, and brace structures to support those components. All outlet tank parts will be labeled with the prefix “OT-” in the CAD files. The outlet tank and corresponding parts list are shown in Fig. 9.

In order to create the hole in part OT-B for the bulkhead fitting to enter, you must rout the hole using the bulkhead fitting hole template previously discussed in the fabrication tools Section 5.2. To do so, begin by securing the cut-to-size OT-B part to the laser-cut bulkhead fitting hole template. Follow the routing procedures outlined in Sections 5.4, 5.6.1 for routing the bulkhead fitting hole in part IN-S3.

To rout the flange shape on the outlet tank wall that meets the test section (OT-S3), use the outlet section flange template. Follow the same routing procedures as outlined in Section 5.4.

Once the bulkhead fitting hole is routed into OT-B, and the OT-S3 is routed to its intended geometry, you may begin welding. The first pieces that should be bonded are the internal spacers and braces to their respective walls. This includes welding OT-S1-A to its wall, OT-S1, gluing OT-S2-A to OT-S2, and gluing OT-S4-C1 and OT-S4-C2 to their wall (OT-S4). These should be done before the pieces are assembled into the final structure.

#### 5.6.5 Post welding

After welding the sections together, coarsely sand the flange mating faces as needed to remove surface defects. Once sanding is complete, where applicable, drill flange holes using appropriate drill jigs. For the test section flanges, use the flange drill alignment template to identify the center points of the holes to be drilled by aligning the template with the flange face. Once aligned, use a transfer punch to mark the location for holes to be drilled. Using the 1/4” acrylic drill bit, place the bit on the center punch mark and drill corresponding holes. For the outlet flange holes, repeat a similar procedure using the outlet section flange template. To drill the inlet flange holes, repeat a similar procedure using the inlet flange drilling template. Once all cutting, welding, drilling, and routing are complete, remove the protective film from the acrylic using tweezers or a utility knife.

**Fig. 10:**
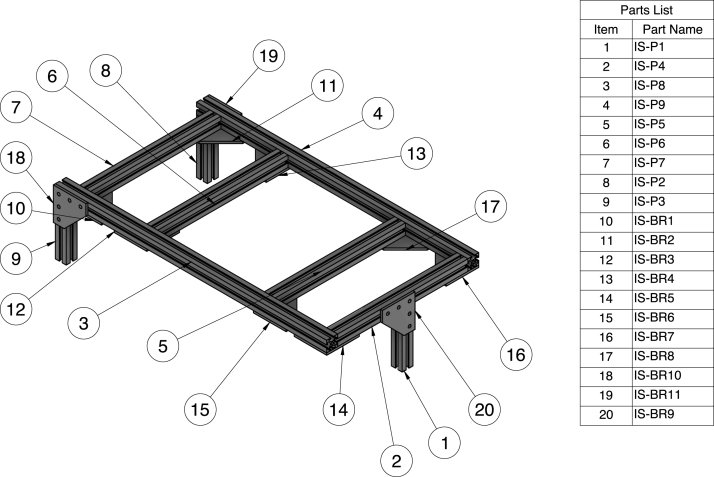
Assembly drawing overview for the portion of the aluminum sub-structure (IS) that supports the inlet tank, constriction, and test section.

**Fig. 11:**
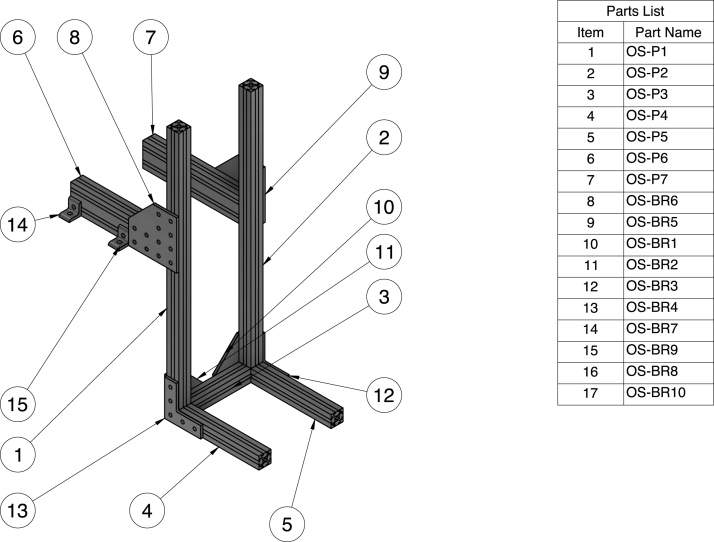
Assembly drawing overview for the portion of the aluminum sub-structure (OS) that supports the outlet tank. Parts 16 & 17 are not labeled but mirror the positions of parts 14 & 15.

### 5.7 Aluminum sub-structures (IS & OS)

The aluminum sub-structure consists of two main sections. The first section (IS) supports the inlet tank, constriction, and test section. This section has three legs for stability and sits on our optical breadboard. The second section (OS) supports the outlet tank and bolts into our optical breadboard for additional support. The aluminum sub-structure is crucial for ensuring a stable and level system that can withstand the weight of the water in the system and minimize warping over time. The sub-structures are shown in Fig. 10, Fig. 11. Assemble the sub-structures per the instructional video available in the digital repository. We ordered pre-cut 80/20 aluminum extrusions but it is also possible to order uncut aluminum extrusion and cut the pieces to length based on the CAD dimensions. When assembling, it is crucial that the supports are level and joined at proper right angles. To ensure proper leveling when assembling the substructure, we used reliable 3 × 2 × 1 blocks when tightening and making adjustments to the structure.

Once the aluminum sub-structures are assembled, you can place the flume body parts on the sub-structure and connect the modular flume body parts to each other as seen in Fig. 4. Use the M5 × 45 mm screws to connect the flume body components, using a washer both where the screw head meets the acrylic face and on the back end of the screw followed by a nut. For the screws at the connection between the outlet tank and the test section, use M5 rubber washers to ensure water tightness.

### 5.8 Plumbing

**Fig. 12:**
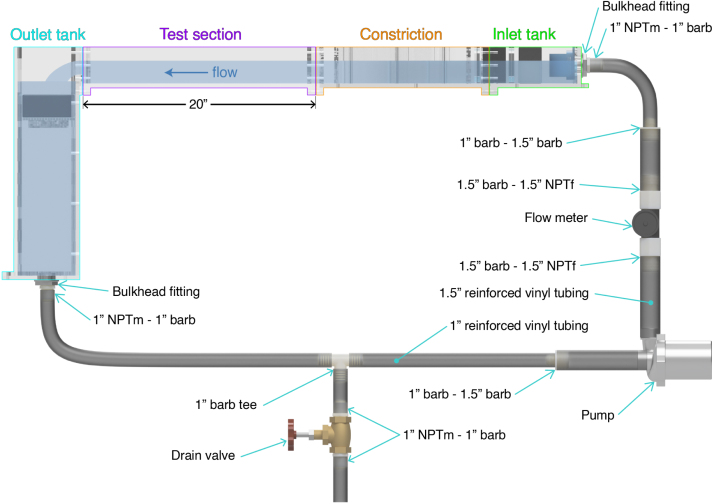
Schematic of the plumbing system with pipe and fitting sizes indicated.

Cut flexible tubing to the needs of your system/work area using a ratcheting flexible pipe cutter. An overall schematic of the plumbing system and tube diameters is shown in Fig. 12.

Beginning at the pump attach the 1.5” tubing to the pump’s outlet. Then, take the flow meter and attach a 1.5” barbed male to 1.5” NPT female to either side of the flow meter. When doing so, wrap Teflon tape along the threads of the flow meter to ensure a water-tight seal. Then ensuring that the arrow of the flow meter is facing up towards the flume system, insert the flow meter with the barbs into the 1.5” tubing. Attach 1.5” tubing to the outlet of the flow meter and a 1.5” to 1” barb. Then attach the 1” tubing. At the inlet tank of the flume body, thread the bulkhead fitting through the hole of the inlet tank. Then thread a 1” barb to 1” NPT male fitting at the outlet of the bulkhead fitting and attach the 1” tubing coming from the pump/flow meter system. Use hose clamps on all barb fittings. Now all your plumbing upstream of the pump is complete.

At the inlet of the pump, attach 1.5” tubing followed by a 1.5” to 1” barb fitting followed by a section of 1” tubing. From there, attach your tee barb fitting. At the section of the tee fitting parallel to the pump, attach a long stretch of 1” tubing. At the outlet tank, install the bulkhead fitting at the hole at the base. Once installed, install a 1” barb to 1” NPT male fitting at the outlet of the bulkhead fitting using Teflon tape on threads. Attach the 1” tubing coming from the pump.

At the section of the tee perpendicular to the pump, attach 1” tubing. Take the NPT gate valve and attach a 1” barb to 1” NPT male fitting on either side of the gate valve, applying Teflon tape at the threads to ensure a water tight seal. Attach the valve with the fittings to the 1” tubing coming off of the tee fitting.

Your plumbing system is now complete. Ensure that all connections are tight, hose clamps have been placed on all barb fittings, and where applicable, Teflon tape is correctly applied.

### 5.9 Flow conditioning & control

There are various measures and accessories for conditioning and controlling the flow within the flume. This section will walk through those measures and detail their fabrication and assembly.

**Table 3:** Overview of flow control components.

Flow control device part name	Materials needed	Tools needed
Inlet diffuser	PLA	3D printer
Inlet diffuser plate	5.5 mm acrylic, M4 × 8 mm screws (qty 5)	Laser cutter, tap set
Filter foam	Filter foam	Utility knife
Honeycomb flow straightener	Straws, super glue	Utility knife
Mesh gate flow conditioners	5.5 mm acrylic, wire mesh screen, M3 × 12 mm screws (qty 4 per gate)	Laser cutter, wire cutter
Channel clamp	9 mm acrylic	Table saw
Weir gates	5.5 mm acrylic	Laser cutter
Diffuser plate	5.5 mm acrylic	Laser cutter

#### 5.9.1 Inlet flow conditioning

**Fig. 13:**
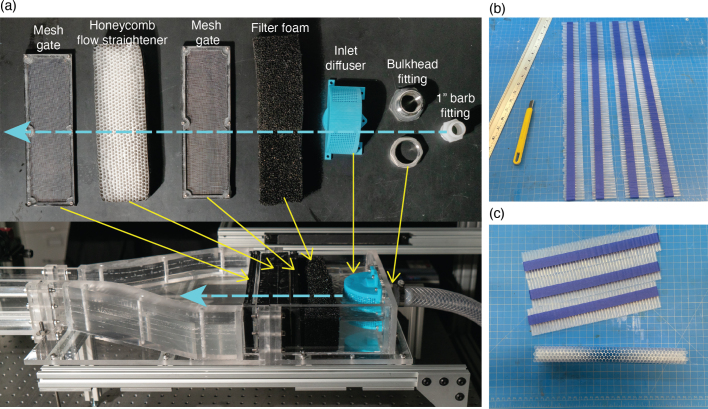
(a) Overview of flow control measures at the flume’s inlet. The flow travels from right to left as indicated by the blue arrow. These measures work to condition the flow as it enters the flume from the pump. (b) Process of cutting straws to equivalent lengths when fabricating the honeycomb flow straightener. Masking tape is used to keep the pieces in place. (c) Gluing process for honeycomb flow straightener fabrication. Each horizontal row is glued one at a time until the straightener is at the desired height.

When the flow enters the flume, it undergoes multiple flow conditioning measures. The plumbing system is connected to the flume via bulkhead fittings. The flow enters from the plumbing system through the bulkhead fitting. The flow then passes through the inlet diffuser, the initial flow conditioning measure, that redirects the incident jet radially out along a half-circle radius. The flow then continues through standard aquarium filter material, followed by a mesh gate, then the honeycomb flow straightener, followed by another mesh gate. These accessories all work to break-up large eddies and straighten the flow that enters turbulently from the pump. Their fabrication methods are outlined below. The individual components and completed inlet flow conditioning assembly are listed in Table 3 and shown in Fig. 13(a).

*Inlet diffuser*: The inlet diffuser is the one component of the flume that is 3D printed. We printed it on a Bambu Labs X1 Carbon Combo 3D printer, but any FDM or SLA printer should work equivalently well. Earlier versions were successfully printed on a consumer-grade MSLA printer. Print the file titled “inlet diffuser” in the CAD file repository, refer to Table 4 for printer settings. To print, orient the diffuser face down, curved side up. Print with sparse supports that can easily be removed. Laser cut the inlet diffuser plate using 5.5 mm acrylic. Tap the five small holes of the laser-cut diffuser plate using a M4 × 0.7 tap. Once the print of the inlet diffuser is complete, remove the supports. To install the inlet diffuser, unscrew the section of the bulkhead fitting on the inside of the inlet tank, leaving the threaded section of the bulkhead fitting. Place the inlet diffuser plate over the threaded section of the bulkhead fitting and align the plate so that it is flush with the top of the tank wall. Screw the previously removed portion of the bulkhead fitting onto the threaded portion of the fitting, securing the inlet plate in the process. Orient the inlet diffuser so that the holes align with those of the secured acrylic plate. Use the M4 × 8 mm screws to secure the inlet diffuser to the inlet diffuser plate.

*Honeycomb flow straightener:* Remove wrappers from plastic straws if necessary. Lay the straws side by side on a piece of masking tape until the straws span the width of the inlet tank. Using a ruler and utility knife, cut all straws to approximately 5 cm using the masking tape to keep the cut ends in place. Using superglue or hot glue, glue the straws, row by row, removing the masking tape once complete. Once the structure is roughly the same height as the inlet wall height of 8 cm, wrap the straw honeycomb structure in vinyl tape to complete the flow straightener. This process is shown in Fig. 13(b, c).

**Table 4:** 3D printer settings for printing inlet diffuser.

3D printer settings used	
Nozzle size	0.4 mm
Layer height	0.2 mm
Number of perimeters	3
Sparse infill density	15%
Supports	Enabled

*Mesh gate:* To make the mesh gates, laser cut two copies of the flow straightener frame using the 5.5 mm acrylic. Tap the holes of the laser-cut pieces using a M4 × 0.7 tap. Cut the woven wire mesh screen roughly larger than the size of the laser-cut pieces, leaving approximately 1/4 inch of mesh on all sides. Sandwich the mesh between the two laser-cut pieces. Using a pen or marker, mark where the holes of the laser-cut pieces align with wire mesh. Remove the mesh and use a utility knife or other method to cut a hole where marked on the mesh screen. Once again, sandwich the mesh between the two laser-cut acrylic pieces and secure by screwing M4 × 12 mm through the initial piece of acrylic, then the mesh, and finally the second piece of acrylic. Repeat for each hole. Cut the excess mesh from the perimeter of the mesh gate. Apply vinyl tape to the edges of the mesh gate to avoid scratches when sliding the gate in and out of the inlet tank.

#### 5.9.2 Channel clamps

The walls of the test section are prone to bowing outwardly, so to prevent this and maintain a consistent width of 50 mm throughout the test section, we designed channel clamps that sit on the top of the channel walls and provide a compression reaction force to maintain a consistent width.

**Fig. 14:**
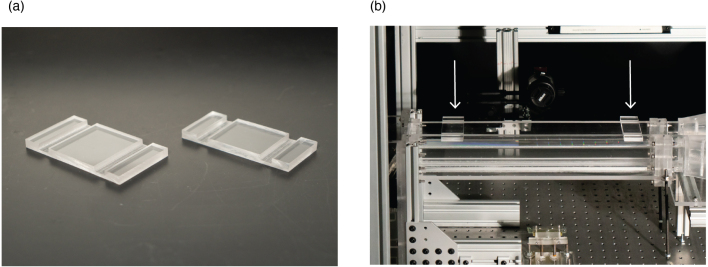
(a) Fabricated channel clamps. (b) Two channel clamps installed along test section walls.

One method for fabricating channel clamps involves using the table saw to make dado cuts to create the slits of the channel clamps. Cut 9 mm thick acrylic to the dimension needed for the channel clamp. The most important dimension for the channel clamp dados is the distance between the outer slit edges as those edges are the ones that will be applying compression to the test section walls. Adjust the table saw blade to cut the dados at a depth of about 4.5 mm. Beginning with the outer slit edges and ensuring that the cuts are made symmetrically, make the first dado cut in the channel clamp. After the critical edge cut is made, you can create the rest of the dado cuts by adjusting your table saw fence to move the dado inward. Since the critical edge is the outer edge of the slits, the width of the dado can vary as long as it is bigger than the thickness of the acrylic test section walls. You want to ensure the total width of the slit is at least 2 mm wider than the thickness of the acrylic of the test section walls. This allows for easier installation of the channel clamps. Sample channel clamps are shown in Fig. 14.

To install the channel clamp, align the channel clamp on the wall of the test section closest to you and tilt the channel clamp towards the other inlet wall until it fits over both walls. Depending on the extent of the bowing, it may be necessary to squeeze the inlet walls together while installing the channel clamps.

#### 5.9.3 Outlet flow conditioning

**Fig. 15:**
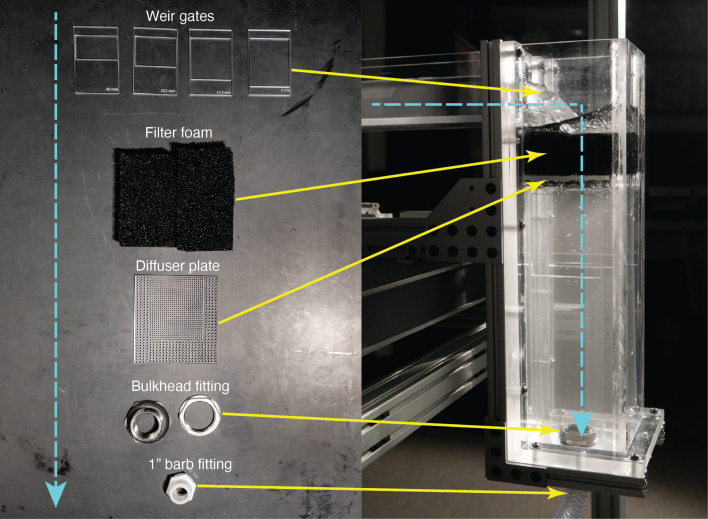
Overview of flow conditioning and control measures at the flume’s outlet. The flow, coming from the test section on the left, travels over the weir and down the outlet tank, as indicated by the blue arrow. The weir works to adjust the water height in the test section while the other measures work to still the outlet flow and thus reduce recirculated bubbles and particles.

After the flow travels through the test section, it experiences multiple flow conditioning measures at the outlet tank as it exits the flume. The flow travels over the weir gate which when combined with the appropriate pump setting (discussed in Section 6) sets the fluid depth in the test section to the targeted depth of 50 mm. Then, the flow enters the outlet tank where it passes through more aquarium filter foam. This filter material is meant to prevent any large particles in the flow from recirculating through the pump to avoid pump damage. Then, the flow passes through a diffuser plate, an acrylic plate with small holes cut throughout that serves as an additional barrier, preventing large particles and entrained air bubbles from recirculating through the flow circuit. The fabrication and installation procedures are outlined below. The individual components and completed outlet flow control and conditioning assembly are shown in Fig. 15.

There are multiple weir gates with differing crest heights, one for each distinct flow velocity. Laser cut the weir gates using the weir gates dxf file. To install, simply slide the weir gates into the weir gate spacer at the outlet flange using the thinner side of the gate as a handle. To install the filter foam, cut the foam to a size that will fit in the outlet tank, resting on the internal ledges of the outlet tank. We used two blocks cut to length in order to fill the space. To fabricate the diffuser plate, use the provided diffuser plate CAD file and cut using 5.5 mm acrylic. Install on the outlet plate internal ledges before placing the filter foam.

### 5.10 Electronics

**Fig. 16:**
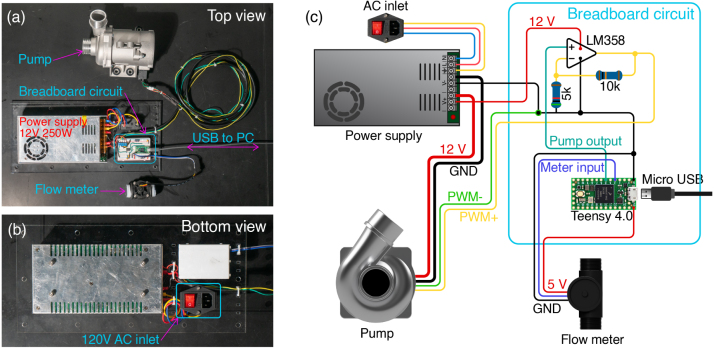
(a) Top view of the electronics system. (b) Bottom view of the electronics system. (c) Circuit diagram of the electronics system.

In this section we describe the components used in the OpenFlume electronics system, as well as the motivation behind each component choice. This guide should be used as a reference for how a control system for OpenFlume can be built and what systems and software worked in our implementation (shown in Fig. 16). Future builders of OpenFlume will readily find alternatives to some of the components used here, which we hope will be informed by the following discussion.

#### 5.10.1 Circuit diagram

The electrical system of the flume can be broken into two subsystems: the power electronics and the control circuitry. The power system runs at 12 V and has a current capacity of 20 A. The control circuitry, consisting of the flow meter and microcontroller, operates at 3.3 V. The microcontroller is used to adjust the pump speed, read the signal from the inline flow meter, and communicate to an external computer. The speed of the pump is controlled by the microcontroller using a pulse-width modulated (PWM) signal. We use a level-shift circuit to boost the 3.3 V pulse signal output from the microcontroller to above the minimum 7.2 V required for the pump PWM+ input.

Several substitutions could be made to the electrical system. For our controller we chose to use a Teensy 4.0 development board. The Teensy is a highly capable microcontroller, and a slower microcontroller would work just as well in this application. The control software requires only that the microcontroller have at least one external interrupt pin and a PWM output. Many alternatives, including the Arduino Uno, can satisfy these requirements. In our implementation, a breadboard circuit consisting of an operational amplifier in a positive feedback configuration is used to level shift the 3.3 V PWM output from the Teensy up to 12 V (Fig. 16(c)). This circuit was designed based on available components in our lab, however it could be replaced by any level-shift circuit capable of shifting 3.3 V to 12 V.

The flume is powered from a 120 V AC outlet. All high-voltage AC wiring should be done with caution by experienced users. The high-voltage AC first passes through a fused, switched receptacle to protect the operator and instrumentation. The line, neutral, and ground wires from the receptacle are attached to their corresponding terminals on the power supply. The power supply specified is a 12 V 250 W switching supply capable of outputting 20.8 A. We first tried a 12 V 120 W supply but found that the available 10 A was not enough for the pump to reach its highest output. Using 14 AWG stranded copper wire with crimped eyelet terminations, we connect the positive and negative power supply rails to the pump power inputs. The same supply rails are used to power our level shifter circuit. Because the power supply output is not earth-referenced, connecting its negative supply rail to the computer’s USB ground does not pose a safety or operational hazard. If the power supply being used does connect the negative output voltage rail to earth ground, care should be taken to ensure it is not possible for a USB connection to the Teensy to cause an internal short as this could damage the flume control circuitry or the computer USB port.

Our flow meter has uses three connections: 5 V, ground, and signal. The 5 V rail is supplied by the 5 V USB power output pin provided by the Teensy. The signal line is connected to a 5 V tolerant external interrupt pin on the Teensy.

**Fig. 17:**
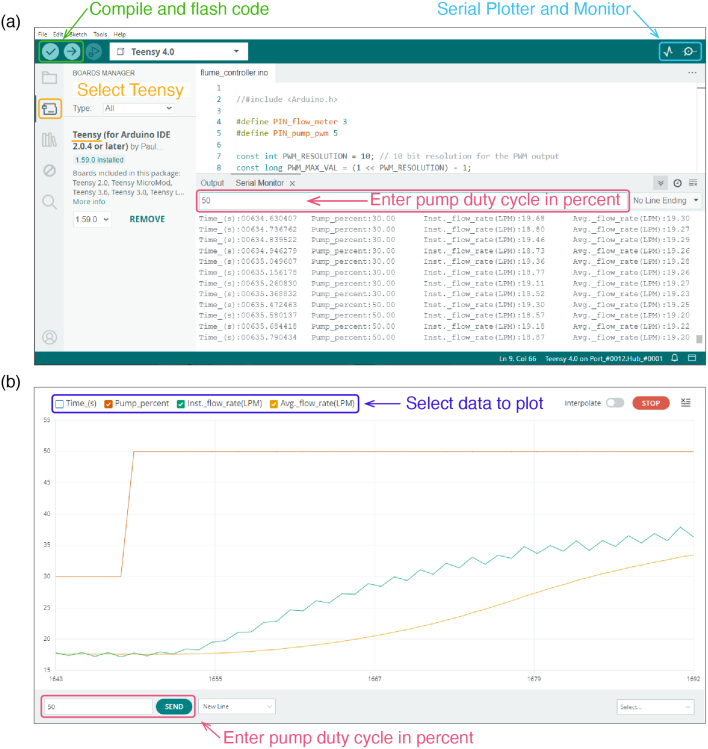
Control interface (Arduino IDE 2.3.3). (a) Microcontroller code can be edited, compiled, and flashed to the Teensy 4.0 using the Arduino development environment. A text-based log of the flume state is displayed in the Serial Monitor (bottom of (a)). A new pump PWM duty cycle percentage can be input in the highlighted text entry field, and sent by pressing enter. (b) The Arduino Serial Plotter is used as a graphical user interface to monitor pump flow rate, PWM percent, and to send new PWM values (bottom left text entry field). It is recommended that the time (‘Time_(s)’) trace be turned off by unchecking the box in the top left, as this trace will continue counting up and thus continuously change the plot scale.

#### 5.10.2 Microcontroller firmware

The firmware running on the Teensy is written in the Arduino programming language. It is responsible for receiving serial input from the user to update the pump speed, measuring the flow meter pulse frequency to determine the flow rate, and reporting these values back to the computer over serial. Since the computer is only used to set the pump and monitor the flow rate, for applications that do not require real-time logging the computer can be replaced by a direct input method to the Teensy. One approach could be the addition of a screen and a knob to adjust the pump set point and display the PWM value and flow meter output. A high level overview of the firmware used in our implementation of OpenFlume is discussed here with commented code available in the repository.

We flash the Teensy using the Arduino integrated development environment (IDE) version 2.3 (shown in Fig. 17(a)). Since the Teensy is not an Arduino branded development board, we must add the support for the Teensy through the board manager. Once added, we can compile and upload the flume_controller.ino file using the check and arrow highlighted in Fig. 17(a). Once flashed, the pump duty cycle and flow rate can be monitored either through the serial monitor which gives a text-based output (Fig. 17(a)) or a graphical output using the serial plotter (Fig. 17(b)).

The CWA-200 pump used in our build of OpenFlume has some idiosyncratic requirements outlined in the pump’s datasheet and also described here. The pump requires a wake-up signal to turn on. To wake the pump, the PWM+ line must be pulsed high for at least 3 ms before starting to send the PWM signal. The PWM frequency must be between 50 Hz and 1000 Hz. According to the datasheet, the pump has a fail-safe where if the PWM duty cycle drops below a minimum threshold value of 12%, the pump will run at 95% of its maximum speed. The pump’s full output speed is achieved at a duty cycle of 97%, above which the pump output speed is reduced to 95%. In our testing, we found that these values are not very accurate. Our pump does not turn on with a PWM value below 15% and seems to increase its flow rate to a maximum at a PWM value of 95% without noticeable drop off between 95% and 100% PWM.

The flow meter documentation provides an equation for calculating the measured flow rate from the output signal. The flow meter has an internal paddle wheel and the rotation of the wheel is measured by an encoder. The output is a square wave where the frequency is directly related to the angular velocity of the internal paddle wheel. To calculate flow rate from the square wave frequency, the provided equation is f=kQ where f is the square wave frequency in Hz, k is a model-specific coefficient, and Q is the volumetric flow rate in liters per minute (LPM). Our flow meter documentation reports the value of k as 0.5 Hz/LPM. We should note that we have not independently verified this value for our particular flow meter. We use the flow meter to monitor drift in the pump output and repeatability of our setup over multi-day experiments but not for precise quantitative measurements.

To calculate the flow rate for our system, we use the equation Q=f/0.5=2f, where again Q is in LPM and f is in Hz. To measure the square wave signal frequency, use the rising edge of the square wave to trigger an interrupt service routine (ISR). In the ISR, the Teensy compares the current and previous rising edge time to measure the signal period, dT, in seconds. The frequency of the square wave in Hz is calculated as 1/dT. To smooth out high-frequency fluctuations, we report the flow meter value as a windowed running average of the last 25 measurements. The size of the averaging window can be adjusted as a parameter in the firmware. The Teensy then reports the percentage of pump power, calculated flow rate, and other state variables via its USB serial connection to the computer. An example of this output can be seen in Fig. 17.

## 6 Operation instructions

1. Fill the flume by adding water to the outlet tank until the water reaches the height of the outlet flange boltheads. Too much water may lead to water spilling over the flume walls and too little water will result in excessive bubbles in the flow.
2. Turn pump’s power supply on.
3. Insert appropriate weir gate, depending on target flow speed. Small gates correspond to higher flow speeds, and vice versa.
4. Using the Arduino serial monitor or serial plotter, enter desired pump power percentage for the given weir gate, typically inputting a value between 15.7–78.4%.
5. Adjust pump power input until the fluid depth in the test section reaches 50 mm.
6. Verify that the water height in the outlet tank is sufficiently high in order to ensure proper stilling and avoid bubbles in the flow circuit. Water level in the outlet tank should be directly below the bolt heads of the weir spacer flange.
7. *Note:* Water level in the outlet tank can also be used to fine tune the depth in the test section. Iterate between adjusting pump power and outlet tank water level to achieve the target depth of 50 mm.
8. To drain, open the draining gate valve, allowing the water to exit the flume system.

## 7 Validation and characterization

### 7.1 Flow velocimetry in test section

In order to characterize the flow in the flume’s test section, we used particle image velocimetry, or PIV. This common technique is an optical method for obtaining the local velocities of a flow. The method works by seeding the flow with tracer particles, illuminating the measurement region with a laser sheet, and capturing images of the illuminated region. The images are taken at a defined time interval and when processing the data, the images are compared between time steps to determine the amount the particles have moved locally in a given time step. From this information, the instantaneous velocities in the field of view can be computed. For this work we used the PIV processing code PIVlab provided available freely [14], with processing parameters and further details discussed in prior work [10]. Images for PIV were collected using an Allied Vision Mako U-130B CMOS camera with 1-MP resolution illuminated by a dual-head Nd:YAG laser (New Wave Solo-I).

We began by determining the highest and lowest flow speed the system could achieve. The lowest achievable speed was determined by the lower limit of the pump (i.e. the lowest PWM at which there was observable flow). The upper limit was constrained by the inlet tank wall height: the highest PWM and thus fastest speed before overflow occurred at the inlet tank. Once we identified the lowest and highest observable flow rates, we iterated on weir heights and pump settings that would allow the water surface in the test section to be 50 mm. We ultimately fabricated seven final weir gates to achieve seven different flow velocities at 50 mm test section depth, spanning from the minimum to the maximum flows that the system could achieve. Measurements of the unsteady free-surface height fluctuations were taken with a laser displacement sensor (Keyence LK-G10) at the center of the channel and were less than 0.2 mm in all cases (standard deviation). The mean heights were within 0.6 mm of the targeted height of 50 mm.

**Fig. 18:**
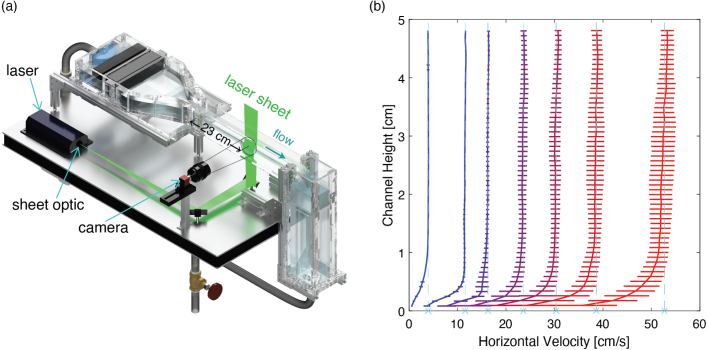
(a) Schematic showing the PIV set-up and the measurement location. (b) PIV results for the streamwise velocity profile along the center of the channel. Error bars indicate the standard deviation of horizontal velocity values resulting from turbulent fluctuations. The dashed vertical lines indicate the estimated free-stream flow speed for each of the seven weir gates tested determined by averaging the horizontal flow speed over the upper 3 cm.

We completed PIV measurements for each of these seven weir heights, calculating the streamwise velocity profile along the center of the channel. We took all PIV measurements at a distance of 23 cm from the test section flange that connects the constriction to the test section. Fig. 18 shows the PIV results for the speed sweep demonstrating the horizontal velocity as a function of the channel height while table Table 5 gives the estimated free-stream velocity for the given weir gate heights. This free-stream velocity is calculated by taking the average horizontal speed of the upper 3 cm of the centerline flow in the test section. The 2D turbulence intensity is also calculated over this same region. This region corresponds to a typical region of interest for running drag experiments in the flume. For all cases, a boundary layer on the bottom of the tank is measured with thickness approximately 1 cm or less. Outside of the boundary layers, the mean flow speed is nearly uniform, as desired.

**Fig. 19:**
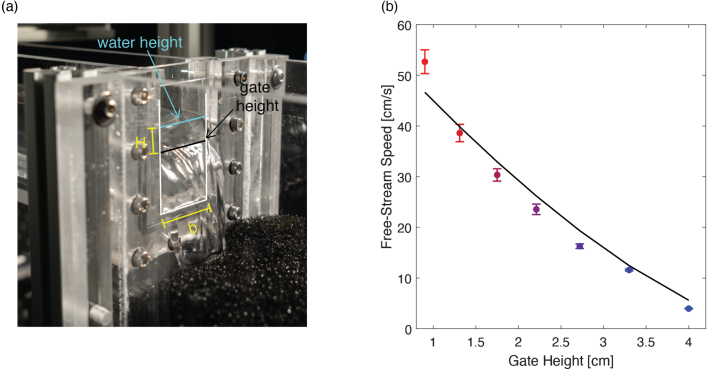
(a) Schematic showing how the variables in the weir equation, Eq. (1), map onto our flume system. (b) Average horizontal velocities experimentally found for each gate height compared to the weir equation. The weir equation is plotted using a fitted curve to estimate a discharge coefficient of $C_{wd}=0.95$ for our system.

We also compared the flow speeds we observed to the well-known weir equation:

(1) $$Q=C_{wd}\frac{2}{3}b\sqrt{2g}H^{3/2}$$

where Q is the discharge flow rate over the weir, b=5 cm is the length of the weir crest, H is the distance between the water surface and the crest (5 cm minus the weir height), and $C_{wd}$ is the weir discharge coefficient determined by the geometry of the weir and other physical parameters. We used a best fit to our experimental data to determine a weir discharge coefficient of 0.95 as shown in Fig. 19, assuming the flow rate Q was equal to the free-stream velocity U times the cross-sectional area of the flow in the test section (25 cm${}^{2}$). Using this empirical value and Eq. (1), one can readily estimate the weir height needed to achieve a target flow speed. The cases characterized here correspond to a range of Froude numbers ($Fr=U/\sqrt{gD}$) of Fr=0.057 to 0.75, where U is the free-stream speed, g is the acceleration of gravity, and D is the fluid depth in the test section (fixed at 5 cm in the present study). Additionally, these cases correspond to a channel Reynolds number ($Re_{D}=\rho UD/\mu$) ranging from $Re_{D}=2000$ to 26,400 where ρ is the fluid density, μ is the fluid viscosity, and D is the characteristic length scale (chosen to correspond to the fluid depth and channel width of 5 cm in our current study).

**Fig. 20:**
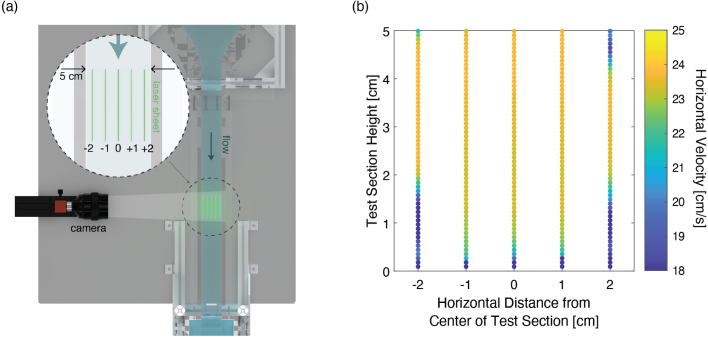
(a) Schematic demonstrating location of PIV measurements for the channel width sweep. (b) Average streamwise velocities across a cross-sectional slice of the test section measuring in the PIV channel width sweep.

We also characterized the streamwise flow profile over the width of the channel at a fixed intermediate speed using PIV. We completed the channel width sweep using the 22.1 mm weir gate corresponding to a free-stream speed of 23.6 cm/s. By measuring the profile at five different locations along the channel width, we were able to plot a sample cross-section of the test section in Fig. 20, where the color of the markers indicates the average horizontal velocity at the corresponding position in the cross-section. Boundaries layers of slower moving fluid are observable along the side and bottom walls, as anticipated. The side wall boundary layers are evidently thinner than the bottom boundary layer, likely a consequence of the quasi-2D constriction geometry (i.e. no geometric contraction in the vertical dimension). Although not explicitly tested here, we anticipate this trend to be consistent for all flow speeds. It is expected that a fully 3D flow contraction would further reduce the boundary layer thickness along the bottom, but at the cost of complicating the manufacturing and assembly process. Furthermore, secondary flow structures are measured in the four corners of the flow cross section, consistent with what is expected for turbulent flow in rectangular ducts [15].

**Table 5:** Measured free-stream speed and turbulent intensity test in test section found using PIV for different weir gate heights. These values are calculated by taking the average of the upper 3 cm of the flow in the center of the test section.

Weir gate height (mm)	Measured free-stream speed (cm/s)	2D Turbulence intensity (%)
9.0	52.7±2.3	4.1
13.1	38.6±1.7	4.0
17.5	30.4±1.2	3.8
22.1	23.6±1.0	4.0
27.2	16.3±0.5	2.6
33.0	11.6±0.2	1.7
40.0	4.0±0.1	1.9

### 7.2 Drag measurement validation

**Fig. 21:**
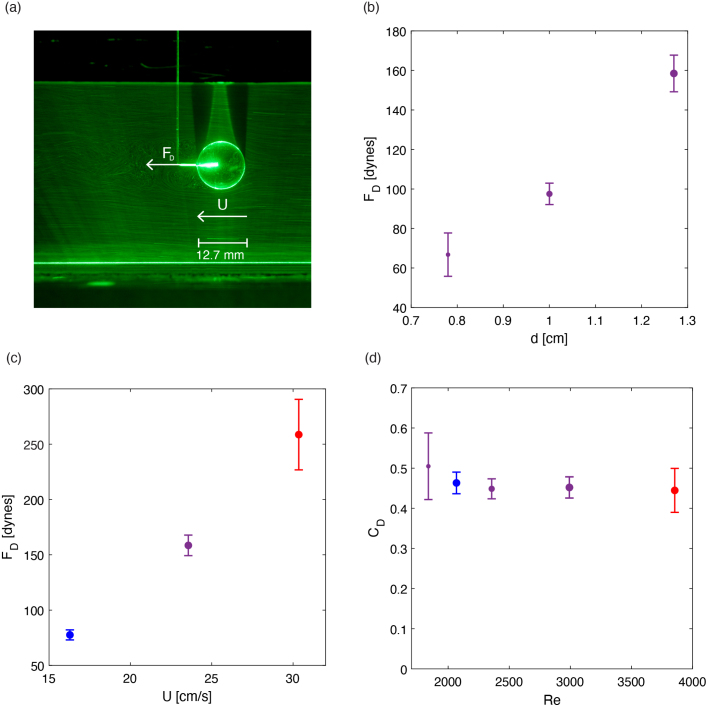
(a) Flow past submerged sphere in the test section. The seeded fluid flow is illuminated from below with a thin laser sheet that intersects the sphere along its central axis. (b) Horizontal drag force $F_{D}$ measurements with respect to sphere diameter d at a constant flow speed U=23.6 cm/s. (c) $F_{D}$ measurements with respect to flow speed at a constant sphere diameter d=1.27 cm. (d) Nondimensional results from both the speed and sphere trials: drag coefficient $C_{D}$ with respect to Reynolds number Re where the color indicates the flowspeed of the trial and the marker size corresponds to the sphere diameter of the trial. Error bars indicate the standard deviation of the force measurement.

In order to further validate the flow of the flume, we examined the force due to drag experienced by a sphere fully submerged at half the depth of the flume’s test section. Using direct measurements of the force of drag on a sphere, we could further test the quality of the flow and validity of the average velocities found in PIV by measuring the drag force on a sphere and comparing it to established empirical values [16]. The non-dimensional drag coefficient is defined as:

(2) $$C_{D}=\frac{2F_{D}}{\rho U^{2}\pi {\left(\frac{d}{2}\right)}^{2}}$$

where $F_{D}$ is the measured drag force, ρ is the fluid density, U is the free-stream velocity, and d is the sphere diameter. This value is well characterized for spheres as a function of the Reynolds number Re=ρUd/μ where μ is the fluid viscosity. For Reynolds numbers between $10^{3}$ and $10^{5}$, the drag coefficient on a sphere is expected to be approximately constant and in the range of $C_{D}=0.4\text{–}0.5$, and thus the dimensional drag force should depend quadratically on both the flow speed and sphere diameter. To conduct the experiment, we attached various spheres of known diameters to a force sensor (Futek Miniature S-Beam Jr. load cell (FSH03867)) via a thin stinger wire as seen in Fig. 21(a), allowing us to directly measure the drag force on the spheres. The force measurement protocol is describe in detail in prior work [10]. In this work, the spheres were positioned in the center of the test section cross-section.

We completed two parametric sweeps for the sphere drag experiment: a size sweep in which the flow speed was constant and we varied the sphere diameter, and a speed sweep where the sphere diameter was constant and the flow speed was varied. The speeds for the sweep were chosen based on the sensitivity of the particular force sensor, as speeds too slow would not induce a force significant enough for the force sensor to accurately resolve. The sizes of the spheres were chosen based on additional experimental constraints such as avoiding the boundary layers of the flume and minimizing potential blockage effects. The results are presented in Fig. 21(b,c) and show the anticipated increase in drag with both radius and flow speed. The corresponding drag coefficients are plotted in Fig. 21(d) with the estimated free-stream velocity extracted from our PIV measurements used to non-dimensionalize the force data. In the range of Reynolds numbers accessible with our flow and force measurement setup, the mean drag coefficient is nearly constant and in the range of $C_{D}=0.4\text{–}0.5$, consistent with historical measurements [16].

### 7.3 Capabilities, limitations, and constraints

As demonstrated via PIV, the flume is capable of providing nearly uniform flows for speeds ranging from 4 to 52 cm/s with boundary layers of less than 1 cm thick on the side and bottom walls. For objects larger than the centimeter scale, a larger test cross-section should be implemented to mitigate potential blockage effects or penetration of the object into the boundary layers. With the current design, the highest achievable speed of the flume is limited by the height of the flume walls rather than the pump’s capabilities. We observe that too high of flow rate (corresponding to high pump PWM) results in overflow at the inlet. Should modifications be made to the overall flume body in the future, higher speeds could be obtained with the existing pump.

Furthermore, there are several observed limitations of the current design that could be addressed in future iterations. For one, we have observed significant bowing of the test section walls over time. When initially fabricated, the channel width when measured at the top of the test section walls was 50 mm. With the channel clamps removed, the maximum width has increased to about 53 mm approximately four months post-fabrication. We have addressed this challenge with the fabrication and use of channel clamps that constrain the width of the test section at the top of the walls to 50 mm. However, future iterations could address this progressive bowing effect without the use of additional clamps or using even thicker acrylic for the side walls.

We have also observed visible standing waves across the length of the test section for higher flow rates. These standing waves became observable for our two fastest speeds measured, 38.6 cm/s and 52.7 cm/s, corresponding to gate heights of 13.1 mm and 9 mm respectively. For all experimental runs, in order to maintain consistency, we measured the depth of the fluid at 23 cm from the test section flange that connects the constriction to the test section. For the fastest speed tested (9 mm weir gate), this location happened to correspond with the local minimum of the standing wave. To achieve the desired height of 50 mm at that location, we had to ramp up the pump power, likely resulting in the slight disagreement with the weir equation expectation as shown in Fig. 19. Further flow and free surface conditioning may remedy these waves in future iterations.

Our final limitation in the design of the system is filtration capabilities. Our current design involves the combination of aquarium bio-sponge filter media and a diffuser plate to both limit the circulation of bubbles and filter out large particles from entering the pump. Nevertheless, with time, the aquarium filter degrades and itself sheds particles with the potential to damage the pump. Thus, an improved filtration system would prevent particles from recirculating while not physically degrading over time. Other filter material choices should be explored in future iterations.

While the current flume was designed, characterized, and validated for hydrodynamic drag experiments, other important applications such as environmental river flows may benefit from future iterations on the device. For instance, adjustable bed slope is an important parameter is such applications, and while modest leveling is currently possible via the aluminum sub-structure, future work might integrate a more intentional and finely tuned bed leveling mechanism into the design.

## CRediT authorship contribution statement

**Maya Lewis:** Writing – original draft, Visualization, Investigation. **Eli Silver:** Writing – original draft, Visualization, Software, Investigation, Conceptualization. **Robert Hunt:** Writing – review & editing, Visualization, Investigation, Formal analysis, Conceptualization. **Daniel M. Harris:** Writing – review & editing, Supervision, Funding acquisition, Conceptualization.

## Declaration of competing interest

The authors declare that they have no known competing interests.
